# Modulating Alginate Hydrogels for Improved Biological Performance as Cellular 3D Microenvironments

**DOI:** 10.3389/fbioe.2020.00665

**Published:** 2020-06-30

**Authors:** Mariana Isabel Neves, Lorenzo Moroni, Cristina Carvalho Barrias

**Affiliations:** ^1^i3S - Instituto de Investigação e Inovação em Saúde, Universidade do Porto, Porto, Portugal; ^2^INEB – Instituto de Engenharia Biomédica, Universidade do Porto, Porto, Portugal; ^3^FEUP - Faculdade de Engenharia da Universidade do Porto, Porto, Portugal; ^4^MERLN Institute for Technology-Inspired Regenerative Medicine, Maastricht University, Maastricht, Netherlands; ^5^CNR NANOTEC - Institute of Nanotechnology, Università del Salento, Lecce, Italy; ^6^ICBAS - Instituto de Ciências Biomédicas Abel Salazar, Universidade do Porto, Porto, Portugal

**Keywords:** alginate, biomaterial, biofunctionalization, 3D cell culture, 4D systems

## Abstract

The rational choice and design of biomaterials for biomedical applications is crucial for successful *in vitro* and *in vivo* strategies, ultimately dictating their performance and potential clinical applications. Alginate, a marine-derived polysaccharide obtained from seaweeds, is one of the most widely used polymers in the biomedical field, particularly to build three dimensional (3D) systems for *in vitro* culture and *in vivo* delivery of cells. Despite their biocompatibility, alginate hydrogels often require modifications to improve their biological activity, namely via inclusion of mammalian cell-interactive domains and fine-tuning of mechanical properties. These modifications enable the addition of new features for greater versatility and control over alginate-based systems, extending the plethora of applications and procedures where they can be used. Additionally, hybrid systems based on alginate combination with other components can also be explored to improve the mimicry of extracellular microenvironments and their dynamics. This review provides an overview on alginate properties and current clinical applications, along with different strategies that have been reported to improve alginate hydrogels performance as 3D matrices and 4D dynamic systems.

## Introduction

Alginate is a natural, marine-derived polysaccharide widely used in the food industry and, more recently, in the biomedical field. In food industry, alginate is used as thickener, texturizer, and stabilizer. More recently, alginate has also been explored for the development of “functional” food (Qin et al., 2018; Bambace et al., 2019), to improve delivery of bioactive compounds (Lupo et al., 2015), as additive of foods and beverages to increase satiety, modulating appetite, glycemia or insulinemia (El Khoury et al., 2014), and in the development of edible food coatings and films (Bambace et al., 2019; Reyes-Avalos et al., 2019) for food packaging applications (Senturk Parreidt et al., 2018).

In biomedical applications, alginate and their hydrogels have been explored, either alone or in combination with other materials, mainly in drug delivery (Nair et al., 2014; Garcia-Astrain and Averous, 2018; Rossi et al., 2018; Shtenberg et al., 2018), tissue regeneration and wound healing (Bidarra et al., 2014; Liu Q. et al., 2017; Luo Z. et al., 2018; Zeyang et al., 2018; Campiglio et al., 2020), three dimensional (3D)-printing (Liu H. et al., 2017; Luo Y. et al., 2018; Wang et al., 2018) and *in vitro* modeling (Cavo et al., 2018; Chu et al., 2018). For tissue engineering, in particular, alginate-based biomaterials have been applied in the repair of both soft and hard tissues, including skin (Han et al., 2017), heart (Sapir et al., 2011; Hayoun-Neeman et al., 2019), bone (Maia et al., 2014a,c), cartilage (Lee H. P. et al., 2017; Liao et al., 2017; Jin and Kim, 2018) and vascular tissue (Bidarra et al., 2011; Torres et al., 2018, 2020), among others. In these fields, chemical modification of polymer-based biomaterials is a frequently used strategy to improve not only their structural and mechanical properties but also their biological activity. Despite being biocompatible, alginate promotes very low protein adsorption due to its high hydrophilicity, being therefore considered a non-fouling material (Morra and Cassineli, 1999). This high hydrophilicity, along with the absence of cell-interactive domains, make alginate hydrogels a non-adhesive biomaterial, as cells cannot establish specific attachment points with the polymer itself. Still, instead of disadvantageous, this characteristic can be useful in biomaterials design, as non-modified alginate can play the role of a blank slate, and be chemically modified to promote specific biological responses in a highly controlled way (Lee and Mooney, 2012).

This review focuses on the use of alginate to develop biomaterials, with emphasis in its application and design to create cellular 3D microenvironments with improved biological performance. First, a brief description on alginate structure and properties will be given followed by examples of alginate-based applications in clinics. Then, alginate modifications for developing hydrogel-based 3D matrices with modulated biochemical and mechanical properties will be explored, including strategies for creating stimuli responsive dynamic systems, also referred to as 4D systems.

## Overview of Alginate as Biomaterial

### Composition, Structural Properties, and Gel-Forming Ability

**Figure 1:**
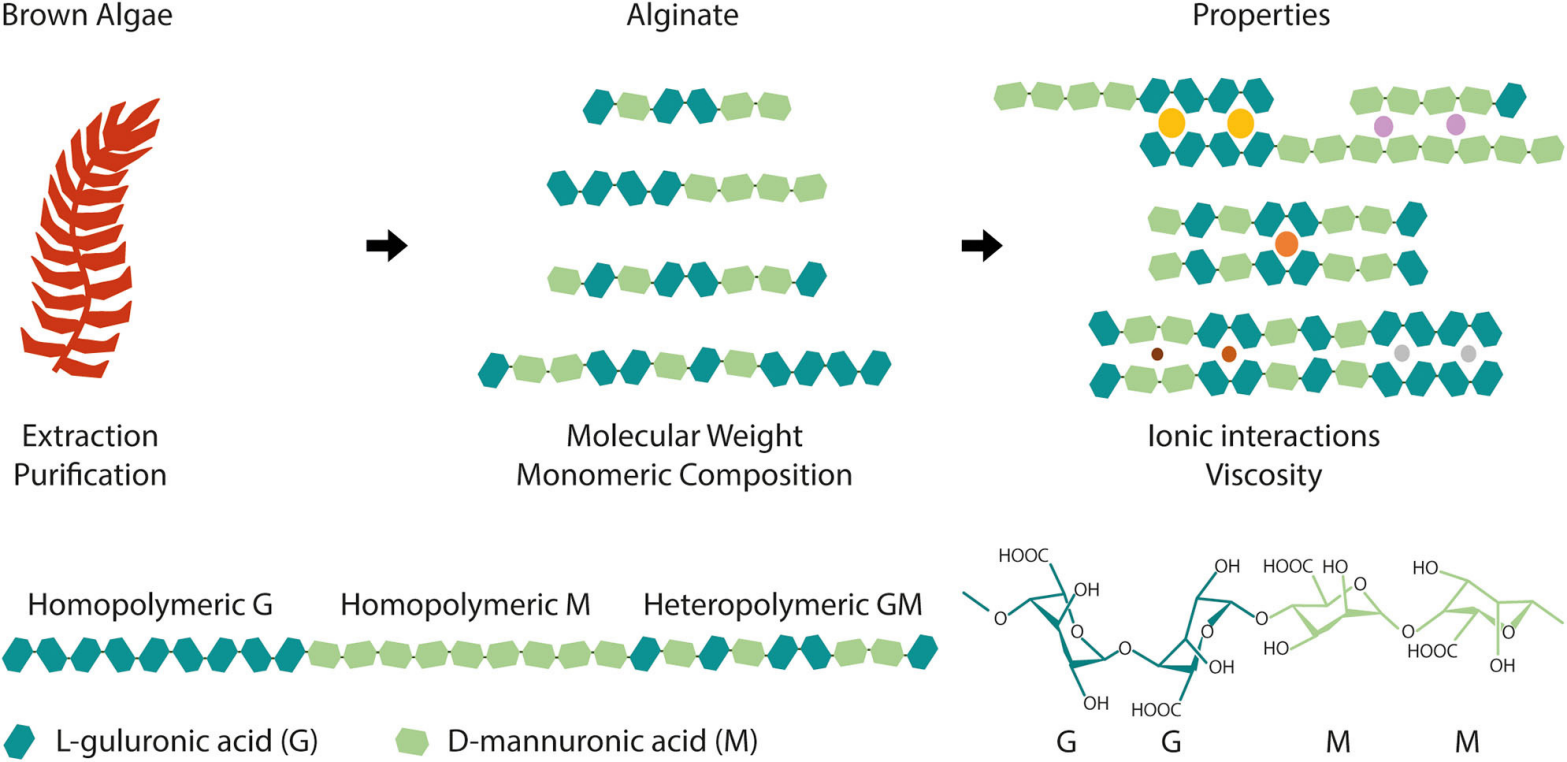
Alginate is extracted and purified from a wide variety of brown algae and it is composed of α-L-guluronic acid (G) and β-D-mannuronic acid (M) blocks. Depending on the species, extraction and purification, alginate can have distinct molecular weight and monomeric composition, namely different G and M blocks content and arrangement (homopolymeric or heteropolymeric regions). These structural differences translate into different material properties, such as alginate affinity toward ions (colored circles exemplify cations with varying radius) and viscosity that will ultimately condition biomaterial development and dictate its final properties and applications.

Alginate is an anionic polysaccharide present in the cell wall of different species of brown algae. Its linear chains contain repeating monomeric units of α-L-guluronic acid (G) blocks and 1,4-linked β-D-mannuronic acid (M) epimers, bearing free functional hydroxyl (OH) and carboxyl (COOH) groups (Figure 1). The relative amount of these monomers (M/G ratio) and their arrangement, either as homopolymeric (GG or MM) or heteropolymeric (GM) blocks, as well as the molecular weight of polymer chains, are highly dependent on the alginate source, regional and seasonal conditions and extraction processes (Haug and Larsen, 1966; Borazjani et al., 2017; Rhein-Knudsen et al., 2017).

Regarding biomedical applications, one attractive characteristic of alginate is its ability to interact with divalent cations (e.g., Ca^2+^), producing hydrogels (polymeric 3D meshes capable of retaining large levels of water) under mild, biocompatible conditions. In this process, alginate undergoes ionic gelation as a result of interactions between divalent cations and alginate free carboxylate (COO^−^) groups, particularly, but not exclusively, those present in G blocks. This ionic gelation process has been previously described by the “egg-box” model (Grant et al., 1973; Braccini et al., 1999; Braccini and Perez, 2001).

Besides Ca^2+^, other divalent cations such as Ba^2+^, Mn^2+^, Sr^2+^, Zn^2+^, and Mg^2+^ may also trigger alginate crosslinking, but with differing affinities (Topuz et al., 2012; Harper et al., 2014). Particularly, Ba^2+^ and Sr^2+^ present higher affinity toward alginate than Ca^2+^ (Kohn, 1975; Siew et al., 2005; Harper et al., 2014). Additionally, trivalent cations of iron (Fe^3+^) are also described to be able to ionically interact with alginate (Sreeram et al., 2004), presenting more affinity than their divalent counterparts (Fe^2+^).

Alginate intrinsic features differently affect the ionic crosslinking process. For instance, ionic interactions with cations is greatly dependent on alginate block composition. Morch et al. (2006) reported distinct cation G/M-dependent binding affinity using alginate microbeads. The authors employed polyuronates to mimic the G, M, and GM blocks of alginate, and studied gelation upon exposure to Ca^2+^, Ba^2+^, or Sr^2+^ (Morch et al., 2006). Whereas Ca^2+^ was able to coordinate with GG and MG sequences, the same was not observed for MM sequences (Morch et al., 2006). On the other hand, Ba^2+^ was able to interact mainly with GG or MM sequences but not with MG sequences (Morch et al., 2006). Finally, Sr^2+^ was able to mainly interact with GG sequences, minimally with MG sequences and not with MM sequences (Morch et al., 2006).

Also, while alginate molecular weight can affect the viscosity and gelation kinetics of alginate solutions, the type, amount and length of G/M block affects the gelation process itself (Braccini et al., 1999; Braccini and Perez, 2001; Fernández Farrés and Norton, 2014; Jensen et al., 2015). In fact, a minimum number of consecutive G blocks is proposed to be required for a junction of the “egg-box” to form (Stokke et al., 1991). The fraction of heteropolymeric (GM) or homopolymeric (GG or MM) regions in alginate also affects gel strength. Alginates with high fraction of heteropolymeric regions produce gels with lower mechanical strength than alginates with high fraction of homopolymeric regions (Draget et al., 1994), which stabilize intermolecular bonds. This can be partially perceived by the structural implications of G and M stereoisomers block content in alginate chains, as schematically represented in Figure 1, where heteropolymeric regions are shown to sterically hinder the packing of adjacent chains and, thus, the formation of junction zones.

These processes can also be affected by external factors. For example, the presence of Na^+^ can affect the viscosity and gelation of alginate solutions and the mechanical properties of ionic hydrogels. Harper et al. (2014) observed that NaCl increases the viscosity of alginate solutions most likely due to increased interpolymer interactions via charge shielding. Additionally, the authors observed a significant decrease in the tensile strength, force to puncture and work to puncture of alginate hydrogel films formed with Ca^2+^, Ba^2+^, Sr^2+^, Zn^2+^ in the presence of NaCl, as compared to films in the absence of NaCl (Harper et al., 2014). These may result from Na^+^ competition with the divalent cations, disrupting some junction zones and producing weaker gels. Temperature also has an impact in the gelation rate and final elastic modulus of ionically crosslinked alginate hydrogels (Moe et al., 1992), with increasing temperatures leading to faster gelation (Kuo and Ma, 2001). The pH interferes with gelation by altering the protonation state of carboxyl groups in alginate chains. These become charged, and thus repulsive at higher pH, destabilizing the hydrogel network, while at acidic pH intermolecular hydrogen bonds stabilize the network as carboxylate groups become protonated. In fact, besides ionic crosslinking, alginate hydrogels can be produced in acidic environments via hydrogen bonding. Such alginic acid hydrogels can be obtained either by acidifying an alginate solution or by exchanging ions with protons in a pre-formed ionically crosslinked alginate gel (Draget et al., 1994).

### Alginate Purification

In order to be used in biomedical applications, careful extraction and purification processes are required to ensure safety of alginate products. Common alginate contaminants include proteins, polyphenolic compounds, lipopolysaccharides (endotoxins) and genetic material (DNA and RNA), some of which may trigger undesirable host responses. Several works have compared the performance of purified vs. unpurified alginates regarding biological properties, particularly the possibility of eliciting undesirable immunological response (Dusseault et al., 2006; Tam et al., 2006; Sondermeijer et al., 2016; Torres et al., 2019). Despite the much lower levels of contaminants in purified alginates comparing to unpurified ones (Sondermeijer et al., 2016), purification is still one of the roadblocks for translational application of alginate-based products to the clinics (Krishnan et al., 2017). Fortunately, alginates of ultrapure grade are already commercially available, with well-defined compositions (i.e., G/M ratio, molecular weight/viscosity), and can be even acquired as sterilized products, if desired (Bidarra et al., 2014).

### Alginate in the Clinics

**Figure 2:**
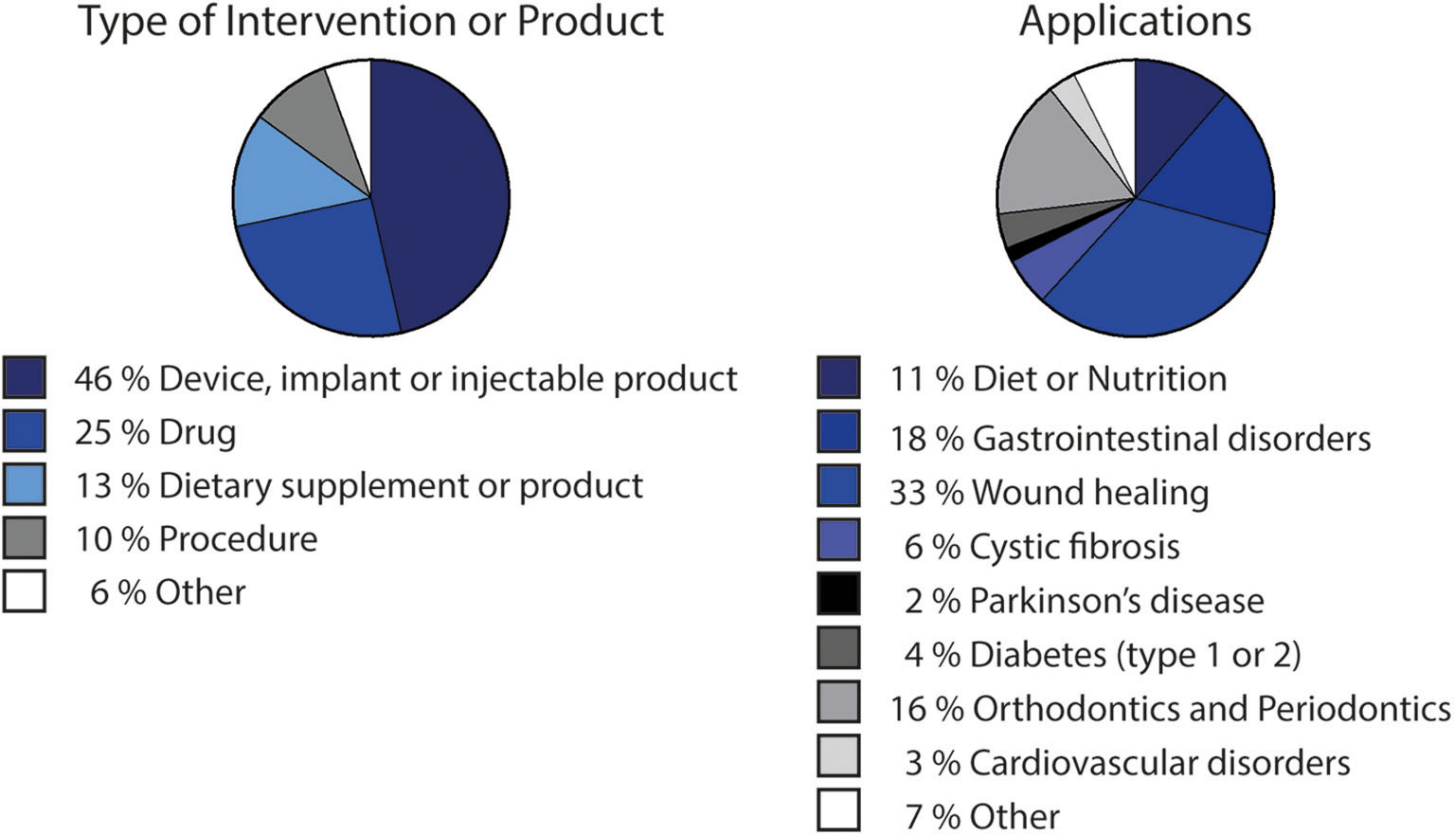
Clinical trials involving alginate-based products or procedures, divided by type of intervention or product and by application. Data adapted from search results matching “alginate” term at ClinicalTrials.gov (2019), i.e., including clinical trials that use alginate-based products/procedures/devices for sake of comparison as standard procedures. Chart showing type of intervention or product considers “Other” matches defined also as “other,” “biological” or “behavioral” by the platform. Applications chart considers applications occurring more than once.

Alginate biocompatibility, along with its unique physicochemical properties, have led to its wide utilization in clinical applications. Currently, around 120 clinical trials are a match for the term “alginate” in the U. S. National Library of Medicine (ClinicalTrials.gov, 2019), with more than half of these studies being considered “completed.” While many of these studies (Figure 2) relate to the use of alginate as dietary supplement, it has also been largely used in drug development. Alginate-based drugs are being studied for the treatment of different types of diseases, such as cystic fibrosis (OligoG by AlgiPharma AS) (AlgiPharma, 2019) or osteosarcomas (Chen et al., 2017). In biomedical applications, most of the matching results correspond to clinical trials of devices, and injectable or implantable products (Figure 2). Such is the case of alginate wound dressings [such as Restore Calcium Alginate Dressing —Silver (Hollister), AqualCel Ag Dressing (Convatec), Algidex Ag® (DeRoyal)], frequently used in clinical trials and probably the most typical commercially available alginate-based biomedical device. Alginate has also been applied in contact lenses (Chong et al., 2016), or as material for dental impression (Ismail et al., 2010). Injectable alginate-based materials are also being developed, such as Algisyl-LVR™ (LoneStar Heart, Inc.), developed for left ventricular augmentation and restoration for patients with dilated cardiomyopathy (Lee et al., 2015; Sack et al., 2018).

Some clinical applications regarding the specific use of alginate-based products as 3D cellular microenvironments present promising results. In xenotransplantation settings, implantation of cells from a different species in humans presents high risk of immunological rejection (Smith et al., 2018). Also, cell therapies involving free cell delivery often result in low cell survival and retention in diseased tissues, which compromises efficacy (Tong and Yang, 2018). In this context, cell encapsulation provides a suitable strategy for functional cellular xenografts without requiring immunosuppression (Smith et al., 2018). Alginate hydrogels can work as carriers for cell transplantation, protecting cells from the adverse host environment and improving their longevity at target sites. Alginate-based capsules for xenogeneic cell therapy have been explored in pre-clinical and clinical trials for applications such as type 1 diabetes, pain, Parkinson's disease and liver failure (Smith et al., 2018). For example, NTCELL® (Living Cell Technologies, 2019) was developed for xenotransplantation of porcine choroid plexus cells for neural applications. LCT recently completed phase 1 and 2 clinical trials on the safety and efficacy of NTCELL® in patients with Parkinson disease (NCT02683629) and announced a successful result comparing to placebo group, prospecting a possible phase 3 study in the near future (Matsumoto et al., 2016). DIABECELL® (Diatranz Otsuka Ltd) is an alginate-based system for immunoprotection of encapsulated porcine islets for patients with type 1 diabetes mellitus, which underwent several clinical trials (NCT00940173, NCT01736228, NCT01739829). Promising results indicated safety in the use of alginate-encapsulated neonatal porcine islet and improvement in diabetic patients (Matsumoto et al., 2016).

Despite positive outcomes in clinical trials, the strict implementation of regulatory guidelines for the development and application of novel therapies is paramount for greater safety and benefit of patients. The use of “combination products”, where biomaterials are used together with bioactive compounds and/or cells has been increasing overtime. This demands stricter and well-defined guidelines and regulation regarding device classification. For instance, in 2013, EMA released a scientific recommendation regarding the classification of advanced therapy medicinal products (EMA/277458/2013), specifically describing the case of alginate encapsulated porcine pancreatic islet cells for type 1 diabetes mellitus. The multiplicity of available approaches and applications of biomedical products prospects that this will be more frequently required for each particular device.

## Biofunctionalization of Alginate Hydrogels With Cell Instructive/Responsive Peptides

**Figure 3:**
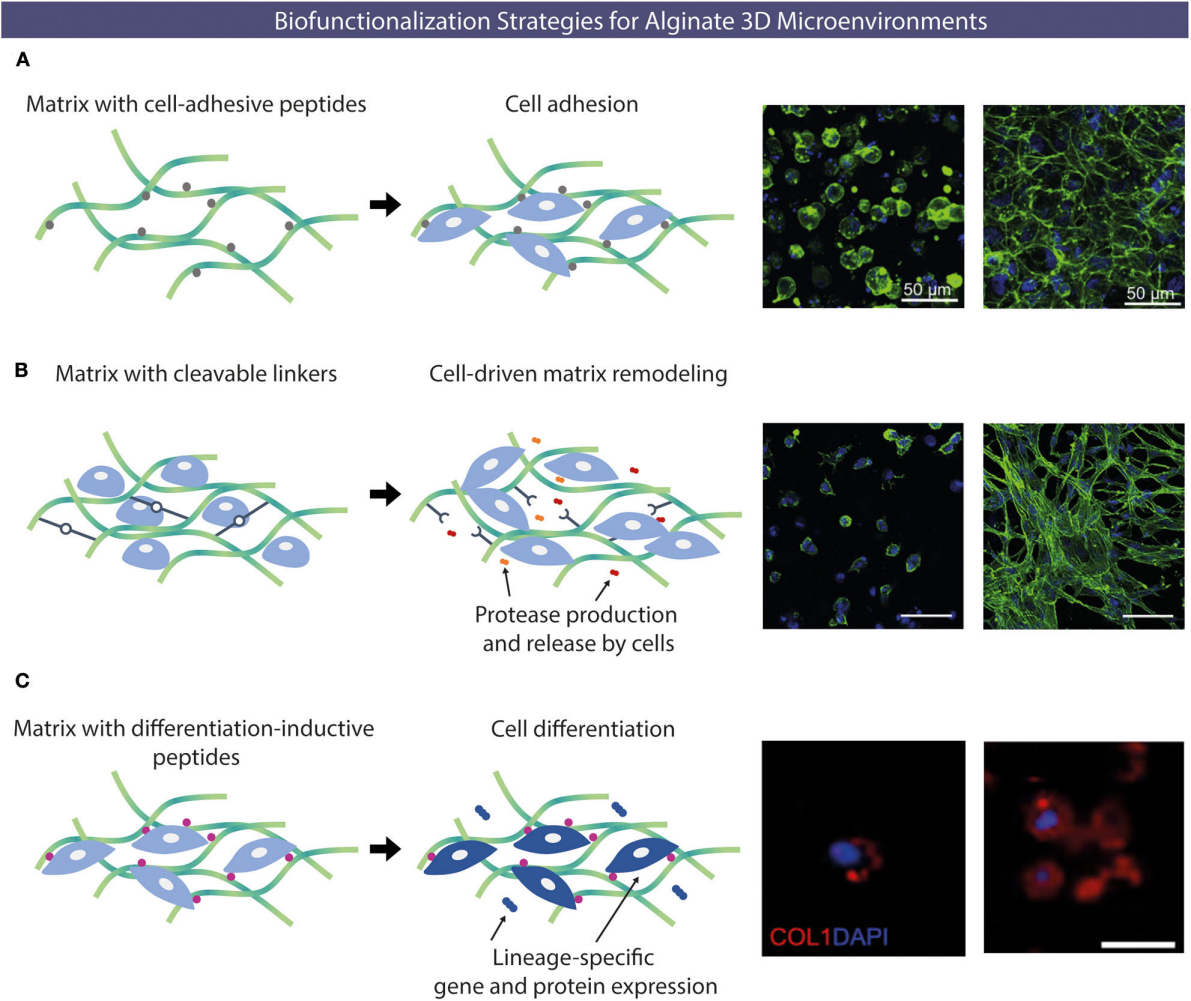
Examples of alginate chemical functionalization with bioactive peptides. **(A)** Cell-adhesion peptides able to interact with different types of cells (e.g., RGD) can be incorporated to promote cell-hydrogel adhesion. Confocal microscopy images of human umbilical vein endothelial cells (HUVECs) cultured under 3D conditions within alginate hydrogels without (left) or with (right) RGD peptides after 48 h (actin in green and nuclei in blue). **(B)** Protease-sensitive sequences peptides can be incorporated, to be degraded upon exposure to proteases (e.g., MMP) produced by cells allowing cell-driven matrix remodeling. Morphology of embryonic mouse fibroblasts entrapped within non-degradable (left) or enzymatically-degradable (right) peptide-crosslinked alginate hydrogels after 14 days (actin in green and nuclei in blue). **(C)** Alginate matrices can also be modified with peptides capable of inducing specific cell differentiation. Collagen type I deposition by MSC transplanted within RGD-alginate (left) and OGP/RGD-alginate (right), showing higher staining intensity for the condition where osteogenic-inductive OGP peptides were incorporated. Different biofunctionalizations strategies can be combined to yield multifunctional hydrogels (collagen type I in red and nuclei in blue). Reproduced/adapted from **(A)** (Bidarra et al., 2011), **(B)** (Fonseca et al., 2011), and **(C)** (Maia et al., 2014a).

Different types of chemical modifications have been performed to increase the versatility of alginate as biomaterials, taking advantage of its native OH or COOH functional groups, using alternative reaction routes, like oxidations (Liu et al., 2018), thiolations (Maleki et al., 2015), reductive-aminations (Rinaudo, 2011; Akhter et al., 2018), sulfations (Kerschenmeyer et al., 2017; Yu et al., 2017), esterifications (Ye et al., 2017), and amidations (Heo et al., 2017; Kondaveeti et al., 2018). Such approaches have been used either as intermediate steps to introduce specific reactive groups for further chemical modifications (Maleki et al., 2015; Liu et al., 2018; Pei et al., 2018), or to directly incorporate new moieties on alginate molecules. This allows to tune different features of alginate polymers and create derivatives with improved properties, such as bioactivity (Kerschenmeyer et al., 2017), mechanical properties (Liu et al., 2018), degradation rate (Fonseca et al., 2014a), fluorescence (Akhter et al., 2018; Araujo et al., 2020), drug loading capacity or release profile (Ye et al., 2017), and antimicrobial activity (Kondaveeti et al., 2018), amongst others. For the design of artificial 3D microenvironments, the incorporation of moieties that specifically modulate cell-material interactions is pivotal. In fact, unlike mammalian polysaccharides such as hyaluronan, a glycosaminoglycan present in the extracellular matrix (ECM) that intrinsically contains functional domains (e.g., cell receptors such as CD44) (Misra et al., 2015), alginate lacks the ability to specifically interact with mammalian cells. To address this, chemical modifications to incorporate cell instructive/responsive moieties in otherwise “bioinert” polymers have been widely performed both in alginate and in several other natural or artificial polymers (Bidarra et al., 2010; Neves et al., 2015; Heo et al., 2017; Zhao et al., 2017; Kudva et al., 2018; Pereira et al., 2018a,b). These “biofunctionalizations” can thus be designed to confer key biological features, like cell adhesiveness (Neves et al., 2015; Zhao et al., 2017; Kudva et al., 2018) or sensitivity to proteolytic degradation (Fonseca et al., 2011; Pereira et al., 2018b), in polymers that despite being biocompatible are inert, non-fouling or non-adhesive materials (Figure 3). Frequently, these bioactive moieties comprise peptide sequences known to specifically interact with cells or cell-derived components. In Table 1 a summary of some peptide sequences that have already been grafted to alginate is presented, and some selected examples will be discussed in detail in the following subsections, which are typical and illustrative rather than all-inclusive.

**Table 1:** Peptide sequences covalently linked to alginate.

**Parental molecule(s)**	**Peptide (sequence)**	**References**
**Fibronectin and Vitronectin**		
RGD-containing	GRGDSP	Formo et al., 2015; Dalheim et al., 2016
	CGGGRGDS	Ooi et al., 2018
	GGGGRGDSP	Bidarra et al., 2010, 2011; Nakaoka et al., 2013; Fonseca et al., 2014a; Maia et al., 2014a,b; Desai et al., 2015; Lee and Lee, 2017
	GRGDY	Rowley et al., 1999
	GRGDYP	Dalheim et al., 2016
	GGGGRGDY	Rowley and Mooney, 2002; Hayoun-Neeman et al., 2019
	AAAAAAKRGDY, VVVVVVKRGDY, GGGGGGKRGDY	Ochbaum and Bitton, 2017
	RGDfK (cyclic)	Sondermeijer et al., 2018
Heparin binding-peptide	GGGGSPPRRARVTY	Sapir et al., 2011; Hayoun-Neeman et al., 2019
PHSRN-containing	GGGGPHSRN	Nakaoka et al., 2013
REDV-containing (from Fn CS5 domain)	CGGREDV, GREDV	Wang et al., 2017
**Osteonectin**		
GHK-containing	GGGGHKSP	Jose et al., 2014; Klontzas et al., 2019
**Laminin**		
IKVAV-containing	GIKVAV	Formo et al., 2015
YIGSR-containing	GYIGSR	Formo et al., 2015
	GGGGYIGSR	Lee and Lee, 2017
**Collagens**		
Collagen type I (α1 chain)	GFOGER	Stephan et al., 2015
**Immunoglobulin superfamily**		
NCAM	KHIFSDDSSE	Dalheim et al., 2016
**Cadherin superfamily**		
N-cadherin (LRP5 peptide)	DSCPPSPATERSYFHLFPPPPSPCTDSS	Lee J. W. et al., 2017
**Growth factors**		
BMP-2	GGGGDWIVA, CGKIPKASSVPTELSAISTLYL	Madl et al., 2014
	NSVNSKIPKACCVPTELSAI	Suzuki et al., 2000
BMP-7 (BFP-1 peptide)	GQGFSYPYKAVFSTQ	Luo et al., 2016; Yang et al., 2018
OGP	GGGYGFGG, GGGIVGPLGYGFGG	Maia et al., 2014a
**Matrix metalloproteinases (MMP) sensitive**		
PVGLIG-containing	GGYGPVG↓LIGGK	Fonseca et al., 2011, 2014a
	GGPVG↓LIGGYGFGG	Maia et al., 2014a
PMSMR-containing	GCRDVPMS↓MRGGDRCG	Lueckgen et al., 2019
Type I collagen-derived	GCRDGPQG↓IWGQDRCG	Lueckgen et al., 2019

*BFP-1 (bone forming peptide-1), BMP (bone morphogenic protein), NCAM (neural cell adhesion molecule), OGP (osteogenic growth peptide). Amino acid nomenclature: alanine (A), cysteine (C), aspartic acid (D), glutamic acid (E), phenylalanine (F), glycine (G), isoleucine (I), histidine (H), lysine (K), leucine (L), asparagine (N), proline (P), glutamine (Q), arginine (R), serine (S), threonine (T), valine (V), tyrosine (Y). ↓ denotes cleavable site*.

### Modification With Cell-Adhesive Peptides

A typical functionalization approach to promote integrin-mediated cell adhesion to alginate hydrogels is the incorporation of RGD (arginine-guanidine-aspartate), a tripeptide sequence naturally present in adhesive ECM proteins (e.g., fibronectin, Pierschbacher and Ruoslahti, 1984; laminin, Sasaki et al., 1988, etc.) (Pierschbacher and Ruoslahti, 1984; Farrell and Al-Mondhiry, 1997). Cells can recognize and bind RGD sequences via cell-surface integrin receptors forming adhesion complexes, namely focal adhesions. These complexes connect the cell cytoskeleton with the ECM, promoting cell-matrix crosstalk and triggering important intracellular signaling cascades, involved in key processes such as adhesion, spreading, proliferation, migration, differentiation and mechanotransduction (Wang et al., 1993; Choquet et al., 1997; Schwartz and Assoian, 2001; Nieberler et al., 2017). Rowley et al. (1999) first reported the modification of alginate with RGD via a carbodiimide chemical reaction. In their work, RGD-coupled alginate improved the adhesion, spreading and differentiation of myoblast cells in hydrogels (Rowley et al., 1999). Since then, not only alginate (Rowley et al., 1999; Rowley and Mooney, 2002; Bidarra et al., 2010, 2011; Maia et al., 2014b; Desai et al., 2015) but also other carbohydrate polymers like dextran (Riahi et al., 2017), pectin (Munarin et al., 2011, 2012; Neves et al., 2015; Pereira et al., 2018b) or gellan gum (Ferris et al., 2015) have been successfully grafted with RGD peptide sequences.

Cellular response is known to vary according to the density and patterning of RGD peptides present in the modified polymer, and the characteristics of the polymer itself (Rowley and Mooney, 2002; Riahi et al., 2017; Pereira et al., 2018b). For instance, Rowley *and coworkers* (Rowley and Mooney, 2002) studied the effect of alginate monomeric ratio and RGD density on the cellular behavior of myoblasts, by comparing high G-content and high M-content alginates (Rowley and Mooney, 2002). For the same RGD density, cells seeded onto alginate hydrogels with increasing amounts of G blocks had increased proliferation rates, as well as extensive myoblast fusion and increased levels of muscle creatine kinase activity, indicating that cells were also differentiating (Rowley and Mooney, 2002). On the other hand, the authors observed that cell adhesion, spreading, proliferation, and differentiation were reduced in low RGD densities (1 fmol/cm^2^) and increased significantly at intermediate values (10 fmol/cm^2^) (Rowley and Mooney, 2002). Interesting, the length of linkers interspacing the grafted peptide sequences from the polymer backbone has also been reported to affect cellular response (Lee et al., 2010, 2016), most likely by affecting the orientation/presentation of the peptide sequence, and, thus, its availability to engage in integrin binding events.

Alginate matrices modified with RGD peptide have been used to develop 3D microenvironments for multiple cell types and reported to improve different aspects of the behavior of MSC (Maia et al., 2014b), endothelial cells (EC) (Bidarra et al., 2011), among others, when compared to the correspondent non-modified polymer. For instance, Bidarra et al. (2011) showed that human umbilical vein endothelial cells preserved their viability, along with the ability to proliferate and migrate when entrapped in RGD-alginate hydrogels, whereas in non-modified alginate cells remained round and unable to spread or migrate. Maia et al. (2014b) observed that MSC embedded in soft RGD-alginate hydrogels were able to exert traction forces and pull the modified alginate network, but not its unmodified counterpart, in order to aggregate into a tissue-like structure and produce endogenous ECM. Desai et al. (2015) reported NIH 3T3 fibroblasts adhesion, spreading and formation of cellular branched interconnected networks in alginate hydrogels, which were RGD-dependent, further illustrating the importance of RGD binding in promoting cell-matrix interactions. Significantly, the modification of bioinert hydrogels such as alginate with RGD peptides is often mandatory and it is typically used in combination with other bioactive peptides.

While the RGD sequence is recognized by specific integrins present in the membrane of most cell types, for some applications, it may be desirable to have some level of control over the type of cell adhering to the biomaterial. Therefore, in those cases, peptide sequences with higher affinity toward particular cell types may be used. For instance, specific peptides can be used to selectively promote adhesion of EC, which play a pivotal role in the formation of new blood vessels, i.e., neovascularization, as reported by Wang et al. (2017). The authors modified alginate and gold nanoparticles with an arginine-glutamate-aspartate-valine (REDV) peptide sequence that is recognized by α4β1 integrin, predominantly expressed by EC (Wang et al., 2017). When modified with REDV, both alginate and gold nanoparticles promoted substantial adhesion of EC, but significantly lower rates of fibroblast adhesion (Wang et al., 2017).

### Modification With Proteolytically-Degradable Peptides

*In vivo*, the ability of cells to remodel the ECM surrounding them is crucial to several cellular activities, such as cell proliferation and migration, being central to (patho)physiological events such as wound healing (Xue and Jackson, 2015), neovascularization (Neve et al., 2014; Bishop, 2015), and tumor formation/progression (Mohan et al., 2020), among others. Likewise, when 3D cultured cells are surrounded by dense polymer meshes, their ability to degrade/remodel the polymer matrix dictates their ability to spread, migrate, proliferate and undergo morphogenesis (Fonseca et al., 2014b). The rate at which degradation/remodeling occurs is also important and should ideally be synchronized with the deposition of new cell-derived ECM.

Following this rationale, biomaterials can be chemically modified to include degradation sites for cell-derived enzymes (Fonseca et al., 2013, 2014a,b). Fonseca et al. (2011) modified alginate with RGD and the proline-valine-glycine-leucine-isoleucine-glycine (PVG↓LIG) peptide sequence that is recognized and cleaved by matrix metalloproteinases (MMP) secreted by cells. The authors cultured MSC under 3D conditions in modified alginate hydrogels and the incorporation of PVG↓LIG along with RGD showed to promote cell spreading and formation of multicellular networks, which was not observed in hydrogels containing only RGD-alginate, where cells remained essentially round and dispersed (Fonseca et al., 2011). This suggests that enzymatic-degradation of PVGLIG motifs allowed cells to create paths within the matrix, at the pericellular space, and partially overcome the biophysical resistance offered by the 3D network (Fonseca et al., 2011, 2013). Upon subcutaneous implantation in immunodeficient mice, MSC-laden RGD/PVGLIG alginate hydrogels were invaded by new collagenous tissue and blood vessels and degraded faster than RGD-alginate hydrogels (Fonseca et al., 2014a). The incorporation of PVGLIG domains also promoted the outward migration of transplanted MSC into host tissue, suggesting that MMP-sensitive alginate hydrogels are adequate vehicles for cell delivery (Fonseca et al., 2014a).

Lueckgen et al. (2019) developed a photocrosslinkable alginate-based system using VPMS↓MRGG or GPQG↓IWGQ containing sequences as degradable crosslinkers. These sequences are MMP-cleavable and had been previously studied for the development of proteolytic degradable PEG hydrogels (Patterson and Hubbell, 2010). In the study of Lueckgen et al. (2019), both sequences were flanked by a linker sequence (GCRD-XXX-DRCG) to react with a norbornene-modified alginate through cysteine thiol groups (Lueckgen et al., 2019). Hydrogels produced with both peptide sequences had similar mechanical and rheological properties, such as elastic modulus and swelling ratio, but degradation kinetics was slower for hydrogels with VPMS↓MRGG in comparison to GPQG↓IWGQ crosslinker when exposed to collagenase (Lueckgen et al., 2019). On the other hand, non-degradable hydrogels produced using non-enzymatically cleavable variants of these sequences and presenting similar mechanical properties showed no susceptibility toward this collagenase, validating the bioactivity of the protease-sensitive sequences (Lueckgen et al., 2019). Ultimately, the incorporation of degradable crosslinkers promoted spreading of embedded fibroblasts within these hydrogels whereas in non-degradable counterparts cells remained essentially round even after 14 days (Lueckgen et al., 2019). *In vivo* results showed higher tissue and cell infiltration into degradable hydrogels in comparison to non-degradable hydrogels (Lueckgen et al., 2019), highlighting the importance of including matrix remodeling cues in the biological performance of biomaterial systems.

### Modification With Differentiation-Inductive Peptides

Specific cell-instructive cues can be incorporated into alginate hydrogels to direct specific lineage commitment of entrapped stem cells. For example, an alginate hydrogel functionalized with an osteoinductive peptide has been developed as vehicle for MSC delivery (Maia et al., 2014a). The bioactive region (the amino acid sequence YGFGG) of the osteogenic growth peptide (OGP) was incorporated into the design of two peptides, where it was flanked by a protease-sensitive linker (PVGLIG) or its scrambled sequence, to provide different OGP release rates (Maia et al., 2014a). OGP peptides were grafted to alginate hydrogels by carbodiimide chemistry, and MSC-laden OGP/RGD–alginate hydrogels were subcutaneously implanted in immunocompromised mice (Maia et al., 2014a). Four weeks after implantation, OGP–alginate hydrogels were more degraded and colonized by vascularized connective tissue as compared to the OGP-free control (RGD-alginate) (Maia et al., 2014a). *In vivo*, hydrogel-entrapped MSC were able to proliferate, migrate from the hydrogels, produce endogenous ECM and induce/trigger mineralization. Significantly, hydrogels with OGP were more effective in promoting osteogenic differentiation of transplanted MSC, than the control (Maia et al., 2014a). Overall, the ability of OGP/RGD-alginate hydrogels to direct the fate of transplanted MSC *in situ* was demonstrated, emerging as a potentially useful system to promote bone regeneration (Maia et al., 2014a).

Guidance of MSC fate commitment into the osteogenic lineage has also been explored in alginate hydrogels integrating peptide sequences derived from bone morphogenic proteins (BMP). For example, BMP-2 derived peptides containing DWIVA and KIPKASSVPTELSAISTLYL (BMP-2 knuckle epitope, BMP-2 KE), have been conjugated to alginate (Madl et al., 2014; Oki et al., 2019). Madl et al. (2014) modified alginate with peptides containing RGD and these two sequences, observing a significant increase in alkaline phosphatase (ALP) activity of osteoblasts entrapped within RGD/BMP-2 KE alginate hydrogels, but not significant differences in osteoblasts entrapped in RGD hydrogels or RGD/DWIVA hydrogels (Madl et al., 2014). Similarly, RGD/BMP-2 KE alginate hydrogels were able to promote osteogenesis in MSC, as observed by the upregulation of Smad signaling pathway, osteopontin overexpression and increased mineral deposition, whereas the same response was not observed for RGD/DWIVA hydrogels (Madl et al., 2014). Likewise, Yang et al. (2018) covalently incorporated BMP-7 derived peptide BFP-1 by carbodiimide chemistry into alginate to develop a lyophilized, porous scaffold capable of inducing MSC osteo-differentiation. The authors observed a significant increase in ALP activity and expression of bone-related genes [osteocalcin, collagen 1, runt-related transcription factor 2 (Runx2) and ALP] and proteins (osteocalcin and collagen 1) in MSC seeded onto BFP-1 modified alginate as opposed to pristine alginate scaffolds (Yang et al., 2018).

## Tuning Alginate Hydrogels Mechanical Properties

The modulation of hydrogels mechanical properties is highly relevant, not only to improve their structural and mechanical stability, but also to guide cellular response via mechanotransduction. As referred above, alginate ability to undergo gelation upon exposure to divalent cations makes alginate quite convenient for biomedical applications. However, such physically crosslinked systems may not present the most adequate rheological and mechanical properties regarding the production steps of biomaterial design or even their final application. On the other hand, it is well-established that cells can mechanically sense and process signals provided by their extracellular environment, to make fate decisions (Lee et al., 2013; Chaudhuri et al., 2016; Lou et al., 2018; Stowers et al., 2019), being thus pertinent to modulate the mechanical features of biomaterials accordingly.

### Covalent Crosslinking for Improved Structural/Mechanical Properties

Alginate can be chemically modified for allowing covalent crosslinking to improve structural stability and modulate rheological and/or mechanical properties. For instance, degradation rates can be reduced when compared to ionic hydrogels, where diffusion and loss of crosslinking ions, presence of chelators (e.g., phosphates) and competition with monovalent cations (e.g., Na^+^) typically present in physiological scenarios interferes with hydrogel integrity over time. Moreover, dual crosslinking strategies can still be explored in alginate covalent hydrogels that still enable non-covalent crosslinking.

One of the most frequent chemical modification performed is the incorporation of photosensitive groups, which allow *in situ* covalent crosslinking under light exposure, in the presence of a photoinitiator (Chou and Nicoll, 2009; Jeon et al., 2014; Pereira et al., 2018b). Photocrosslinking strategies are advantageous by providing spatio-temporal control over the crosslinking process, through the tuning of intensity and duration of light exposure, the concentration and type of photoinitiator and the extent/pattern of exposed vs. non-exposed regions. Samorezov et al. (2015) incorporated methacrylate groups in alginate and explored different modification degrees to produce hydrogels that could be either ionically crosslinked, photocrosslinked or both (dual crosslinking) (Samorezov et al., 2015). The authors showed that shear moduli (storage and loss) were significantly increased in dual crosslinked alginates, while the swelling ratio was significantly decreased (Samorezov et al., 2015). By further modifying methacrylate alginate with RGD sequences, the authors showed that pre-osteoblastic cells were able to adhere and spread on-top of both ionically and dual crosslinked hydrogels, while presenting higher proliferation rates in dual crosslinked hydrogels (Samorezov et al., 2015). By controlling the regions exposed to UV light, the authors were also able to create patterned structures with regions of dual crosslinked (exposed regions) or ionically crosslinked regions (non-exposed regions) presenting differences in mechanical properties and cell response (i.e., adhesion and spreading) (Samorezov et al., 2015). Desai et al. (2015) developed covalently-crosslinked alginate by introducing norbornene and tetrazine groups in the polymer backbone by carbodiimide chemistry, which react with each other promoting crosslinking, and posteriorly modified it with a mono-thiol RGD sequence by a thiol-ene photoreaction. Ooi et al. (2018) used thiol-ene click chemistry for reacting norbornene-alginate with thiol-containing polymers (e.g., PEG dithiol or 4-arm PEG thiol) crosslinkers, which enabled better spatio-temporal control of alginate rheological and mechanical properties during bioprinting. The authors were able to tune hydrogel properties by varying the concentration, molecular weight and number of arms (2 or 4) of PEG crosslinker used (Ooi et al., 2018). For instance, hydrogels produced with higher crosslinker concentration, lower molecular weight PEG or 4-arm PEG presented lower swelling ratios, as they form more compact networks (Ooi et al., 2018). On the other hand, the storage moduli increased with increasing crosslinker concentration and number of arms, but hydrogels produced with longer PEG crosslinkers (5000 Da) were slightly stiffer than the ones produced with smaller PEG (1500 Da) (Ooi et al., 2018). By further ionically crosslinking these hydrogels, the authors were able to significantly increase storage and loss modulus (Ooi et al., 2018). Indeed, the possibility of producing alginate derivatives for covalent crosslinking while retaining, even if partially, its ability to undergo ionic crosslinking, is one of the key features increasing the versatility of alginate regarding the tuning of mechanical properties.

Temperature can also be used to catalyze the crosslinking of alginate hydrogels. For instance, Wang et al. (2015) developed a thermal polymerizable modified alginate incorporating glycidyl methacrylate groups. In the presence of a thermal initiator, the modified alginate could be rapidly (5 to 20 min) crosslinked at 37°C (Wang et al., 2015). This type of modification can be of great interest when developing biomaterials for implantation, as these can be crosslinked *in situ*, triggered by normal body temperature, in a rather short period of time.

Covalent alginate hydrogels can also be enzymatically crosslinked. Hou et al. (2015) conjugated alginate with dopamine and tyramine by carbodiimide chemistry, which react in the presence of hydrogen peroxide (H_2_O_2_) and horseradish peroxidase (HRP), promoting crosslinking (Veitch, 2004). In this system, HRP reacts with H_2_O_2_, eventually leading to the formation of catechol or phenol radicals in the dopamine or tyramine molecules, respectively, that then bind to form covalent links. In this case, the mechanical properties and gelation times can be tuned by altering the amount of H_2_O_2_ in the system (Hou et al., 2015). Ganesh et al. (2013) developed a similar system based in tyramine substituted alginate for cell encapsulation and delivery.

### Mechanical Modulation for Specific Cell Guidance

In the development of 3D hydrogel systems, stiffness is probably one of the most routinely characterized mechanical parameters. Indeed, stiffness is known to impact cell adhesion, spreading, proliferation and even differentiation, at different levels, for different cell types (Yeung et al., 2005; Park et al., 2011; Mao et al., 2016). Studies on RGD clustering and traction forces exerted by cells in 2D alginate substrates showed that spreading of preosteoblastic cells was independent on matrix stiffness, but the formation of focal adhesion and cell proliferation was significantly enhanced with increasing substrate rigidity (Kong et al., 2005). The authors proposed that a particular resistance to ligand displacement may be required for cells to initiate the necessary apparatus to generate traction forces, while softer gels may fail to provide sustainable anchor sites, impairing the formation of focal adhesions (Kong et al., 2005). Within 3D alginate hydrogels with varying stiffness, entrapped MSCs were reported to present stiffness-dependent proliferation and differentiation (Huebsch et al., 2010; Maia et al., 2014b). In their work, Maia et al. (2014b) modulated stiffness by varying alginate dry mass content, with the storage modulus of 2 wt% hydrogels being 40-fold higher than 1 wt% hydrogels, and with this latter possessing higher viscous-to-elastic ratio (Maia et al., 2014b). Even though MSCs viability was independent of matrix composition, in softer hydrogels cells were able to proliferate and contract the polymeric network, forming dense multicellular aggregates with extensive cell spreading and cell-cell contact, whereas in stiffer hydrogels MSCs remained round and disperse (Maia et al., 2014b). ECM production was also affected by stiffness, with softer alginate matrices leading to higher fibronectin production than stiffer matrices, where fibronectin remained confined to the intracellular space (Maia et al., 2014b). Also, Maia et al. (2014b) observed that cells were only able to substantially contract the artificial matrix in the presence of RGD (Maia et al., 2014b). Similarly, Huebsch et al. (2010) observed that encapsulated MSC preferably undergo osteogenic differentiation within 11–30 kPa hydrogels, whereas in 2.5–5 kPa hydrogels they tended to undergo adipogenesis (Huebsch et al., 2010). However, when blocking RGD binding to α5 integrins, osteogenesis was significantly impaired and adipogenesis enhanced, showing a partial modulation of cell fate by integrin/adhesion-ligand bond formation (Huebsch et al., 2010). In fact, α5-RGD bonds presented a biphasic dependence on matrix stiffness that peaked at 22 kPa, the optimal value for osteogenic differentiation, whereas on softer and stiffer matrices this cell-RGD bond formation would decrease (Huebsch et al., 2010). Also, it was in 22 kPa matrices that the authors observed the highest traction-mediated matrix reorganization (Huebsch et al., 2010). This suggests that there is a minimum of matrix stiffness required for the formation of sustainable anchor sites, allowing cells to exert traction forces, while in excessively stiff hydrogels the tension exerted by cells may not be sufficient for matrix deformation (Kong et al., 2005; Huebsch et al., 2010).

More recently, it has been acknowledged that stress relaxation, i.e., the time-dependent decrease in stress under a constant strain, also impacts cell behavior both in 2D and 3D systems, including on alginate hydrogels (Chaudhuri et al., 2015, 2016; Bauer et al., 2017; Lee H. P. et al., 2017). Stress relaxing alginate hydrogels are often produced by ionic crosslinking, as physical alginate hydrogels more closely resemble the viscoelastic properties of the native ECM, as opposed to covalent hydrogels which are mainly elastic and present significantly less stress relaxation (Chaudhuri et al., 2016). Stress relaxation observed in physical alginate hydrogels is thought to occur due to the unbinding and rebinding of ionic crosslinkers (Zhao et al., 2010) and can be modulated by varying alginate molecular weight or by covalent incorporation of spacers into the polymer backbone (Chaudhuri et al., 2016; Nam et al., 2019). Decreasing alginate molecular weight leads to enhanced stress relaxation, most likely due to altered connectivity and chain mobility (Chaudhuri et al., 2016). Incorporating spacers onto alginate backbone can enhance stress relaxation, in comparison to unmodified alginate, by sterically impairing ionic crosslinking, as observed by Chaudhuri et al. (2016) who coupled 5 kDa PEG spacers to low molecular weight alginate (35 kDa) (Chaudhuri et al., 2016). Similarly, the length and density of PEG spacers coupled to alginate can be used to modulate stress relaxation independently of elastic modulus, with higher PEG density and length leading to faster stress relaxation rates (Nam et al., 2019).

Interestingly, modulating stress relaxation can be used to guide cell behavior and matrix remodeling, in a stiffness-independent manner. For instance, even though cells spread more when seeded onto stiffer substrates, faster stress relaxation leads to higher cell spreading and stress fiber formation in softer substrates (Chaudhuri et al., 2015). Such effects of stress relaxation are dependent on cell-adhesive peptide density, being enhanced in the presence of high RGD densities and promoting the formation of focal adhesions (Chaudhuri et al., 2015). Cell proliferation and lineage commitment can also be modulated by stress relaxation (Chaudhuri et al., 2015, 2016). Chaudhuri et al. (2016) observed that MSCs differentiation into adipogenic and osteogenic lineages was differently affected by stiffness and stress relaxation (Chaudhuri et al., 2016). MSCs entrapped within 9 kPa hydrogels committed to the adipogenic lineage but presented lower differentiation levels with faster stress relaxation (Chaudhuri et al., 2016). On the other hand, cells within 17 kDa hydrogels which committed to the osteogenic lineage had enhanced differentiation levels within hydrogels with faster stress relaxation (Chaudhuri et al., 2016). Such interference of stress relaxation in cell behavior and fate seems to correlate with local RGD clustering, myosin contractility and localization of the transcriptional factor YAP, known to regulate cellular response to mechanical and geometrical cues, particularly during osteogenesis (Chaudhuri et al., 2016).

Matrix formation by encapsulated chondrocytes is also reported to be affected by stress relaxation in alginate hydrogels with non-relaxing hydrogels leading to matrix deposition adjacent to cells, whereas fast relaxing hydrogels allow higher collagen and aggrecan deposition in an interconnected manner (Lee H. P. et al., 2017). Additionally, the upregulation of anabolic genes (collagen II and aggrecan) within faster relaxing hydrogels is reported, as opposed to slow relaxing hydrogels that promote the upregulation of catabolic genes (ADAMTS4 and MMP13) (Lee H. P. et al., 2017).

Altogether, these studies show the impact of mechanical properties in cell-matrix interactions and, consequently, in the ability of cells to sense, respond and transform the surrounding 3D network. In particular, these findings strength the versatility of alginate hydrogels as 3D microenvironments with tunable mechanical properties, allowing the independent modulation of different parameters such as stiffness and stress relaxation. The intrinsic bioinertness of alginate is a clear advantage in these scenarios, since the presence and density of cell adhesive moieties can be finely controlled, which may elucidate their role in cell-ECM interactions, particularly regarding the mechanical environment. Overall, these examples illustrate the possibility of designing systems for cell guidance via adequate tuning of mechanical cues, alone or in combination with biological cues as the ones presented in the previous section, with improved mimicry of the native ECM.

## Alginate 4D Systems as Dynamic Microenvironments

In native tissues, the ECM is a highly dynamic environment, being constantly altered both under physiological and pathophysiological contexts, not only regarding its biochemical composition but also in terms of mechanical properties. As explored on the previous sections, exposing cells to biological moieties and/or particular mechanical conditions can elicit specific cellular outcomes. However, in most of the reported studies these stimuli were present from the beginning, with cell-material and cell-cell interactions being conditioned right upon culture within these 3D microenvironments. Nevertheless, temporal changes may be relevant for biomaterials performance, and understanding how cells react to certain cues in a specific time point or at a specific biological phase can be of profound interest in the study of cell-microenvironment interplay. Inspired on that, biomaterials design has been evolving into the creation of four-dimensional (4D) systems that are able to dynamically change in response to an applied stimulus, a long time, in a predictable or on-demand manner.

Controlled presentation of biological moieties, as the ones explored in section Biofunctionalization of Alginate Hydrogels with Cell Instructive/Responsive Peptides, in alginate hydrogels has already been reported. One example is the covalent coupling of peptides, *in situ*, after cell encapsulation. Oki et al. (2019) produced an alginate derivative containing maleimide groups, which were able to react under physiological conditions with thiol-containing peptides (Oki et al., 2019). The authors explored this platform to switch cell proliferation and differentiation by encapsulating cells within maleimide-alginate microcapsules and then exposing them to the cell adhesive RGD peptide and the BMP-2 mimetic DWIVA and BMP-2 KE peptides 1 day after encapsulation (Oki et al., 2019). Efficient peptide coupling occurred, without affecting mechanical properties (Oki et al., 2019). On days following RGD coupling, fibroblasts proliferation significantly increased when compared to cells encapsulated within microcapsules where RGD was non-covalently bound or not present (Oki et al., 2019). Similarly, only when BMP-2 mimetic peptides were covalently bound to the alginate, preosteoblastic cells were able to differentiate into osteoblasts (Oki et al., 2019). Even though no other timepoints for peptide coupling were explored in this study, this strategy can be envisioned for dynamic systems were peptide moieties can be coupled in a sequential and timely fashion for guided cell behavior.

Structural/mechanical alterations of alginate hydrogels can also be promoted in a dynamic and controlled fashion for cell guidance. Gillette et al. (2010) developed a hybrid hydrogel combining alginate and collagen I, where the dynamic switch of alginate gelation was used to modulate the structural features of the microenvironment (Gillette et al., 2010). In this case, the structural switch was achieved by exposing hybrid hydrogels to either crosslinking Ca^2+^ ions to reinforce gelation, producing a tighter biomaterial network, or to sodium citrate (Ca^2+^ chelator), producing a more permissive microenvironment with a more open network (Gillette et al., 2010). The authors observed that cell spreading and migration were impaired in matrices where alginate was more heavily crosslinked, but when crosslinking was reverted, cells were able to spread and, interestingly, cell spreading was retained even after alginate recrosslinked (Gillette et al., 2010). Noteworthy, this type of switch affects not only the structural features (i.e., the mesh size), but also the mechanical properties of hydrogels. On one hand, diffusion of biological compound and cell motility may be affected by alteration in the network mesh size. On the other hand, cells may also respond to concomitant changes in matrix stiffness. Thus, dissociating the effect of each individual factor may be challenging.

Temporal control over hydrogel matrix stiffness can also be achieved by Ca^2+^ release from temperature sensitive liposomal vesicles incorporated within alginate hydrogels. In this case, the release of divalent cations from liposomes can be achieved simply by heating (Westhaus and Messersmith, 2001), or in alternative, exposure to near infrared (NIR) light if temperature-sensitive nanoparticles, as gold nanorods, are included within the lipidic vesicles (Stowers et al., 2015, 2017; Joyce et al., 2018). In this latter example, upon NIR light exposure, gold nanorods undergo surface plasmon resonance, increasing local temperature and disrupting the liposomal lipid bilayer, ultimately leading to Ca^2+^ release and matrix stiffening (Stowers et al., 2015). Notably, the same strategy can be used to encapsulate Ca^2+^ chelators within liposomes, which will revert hydrogel crosslinking upon release, promoting matrix softening instead (Stowers et al., 2015). Besides preserving cell viability (Stowers et al., 2015), the use of NIR light can be advantageous for biomedical applications due to its high penetration through biological tissues, when compared to UV light. Indeed, in their work, Stowers et al. (2015) were able to induce matrix stiffness *in vivo* upon transdermal NIR exposure (Stowers et al., 2015). Alginate-liposome hydrogels with dynamic mechanical features have also been used to study the impact of stiffening in the formation of acinar structures by mammary epithelial cells (MEC) (Stowers et al., 2017). Non-malignant MEC were cultured within alginate-matrigel hydrogels and cultured for 14 days to allow for acinar development, being afterwards exposed to NIR light to achieve stiffening until tumor-like moduli (~ 0.5 up to 1 kPa) (Stowers et al., 2017). Interestingly, the authors observed that cells embedded within stiffened gels presented an invasive phenotype with multicellular protrusions and collective cell migration, as well as significantly increased proliferation and acini size, as opposed to cells within non-stiffened matrices, which preserved their original phenotype (Stowers et al., 2017). Studies with dynamic systems such as the ones described herein clearly illustrate that cells effectively respond to dynamic microenvironmental changes. These artificial microenvironments provide better representations of native processes and may improve current knowledge on fundamental biological events. They can also be helpful tools for the development of therapeutic strategies, which can take into consideration alterations in drug resistance by cells, as a result of all phenotypical alterations triggered by the structural changes of the extracellular microenvironment, for example (Joyce et al., 2018). Dynamic photo-induced mechanical modulation can be also used to create mechanical gradients and patterns to assess cell-material and cell-cell interactions under identical culture conditions but structurally distinct local microenvironments (Stowers et al., 2015).

## Conclusions

After its extensive use in food and pharmaceutical industries, alginate clearly settled its potential for biomedical applications. Despite the lack of inherent mammalian cell-interactive domains, the composition and structure of this natural polysaccharide along with its biocompatibility enable the development of biomaterials for a wide range of applications. By fine-tuning different properties, such as the molecular weight, backbone block composition and distribution, polymer concentration, and type/amount of crosslinkers it is possible to modulate the viscoelastic properties of alginate solutions and their hydrogels. The ability of alginates to form hydrogels under mild and biocompatible conditions, is indeed one of its most appealing characteristics, when developing biomaterials for *in vitro* and *in vivo* applications.

Alginate relevance in the biomedical field is certainly potentiated by the possibility of creating different types of derivatives through chemical modifications, to render alginate cell-interactive and and/or modulate its crosslinking mechanisms, as discussed in this review. Such (bio)functionalizations greatly increase the versatility of alginate as a biomaterial, and ultimately reflect on its biological performance. While the field of biomaterials science is continuously evolving through the design of increasingly complex and “smarter” systems, of which dynamic 4D systems are probably the best example, alginate remains one of the most frequently used natural-based polymers.

Collectively, alginate attractive characteristics and safety have surely contributed to the existence of multiple alginate-based products in the biomedical field, and further prospects its potential for more complex and combinatorial therapeutic approaches, as indicated by the ongoing clinical trials.

## Author Contributions

MN conceptualized and wrote the main manuscript and prepared the figures. LM edited and reviewed the manuscript. CB conceptualized, edited, and reviewed the manuscript. All authors approved the submitted version of the manuscript.

## Conflict of Interest

The authors declare that the research was conducted in the absence of any commercial or financial relationships that could be construed as a potential conflict of interest.
